# Haemodynamic characterisation of different endotypes in coronary artery vasospasm in reaction to acetylcholine

**DOI:** 10.1016/j.ijcha.2022.101105

**Published:** 2022-08-13

**Authors:** Rutger G.T. Feenstra, Coen K.M. Boerhout, Caitlin E.M. Vink, Janneke Woudstra, Marianne E. Wittekoek, Guus A. de Waard, Yolande Appelman, Etto C. Eringa, Koen M.J. Marques, Robbert J. de Winter, Tim P. van de Hoef, Marcel A.M. Beijk, Jan J. Piek

**Affiliations:** a Amsterdam UMC, Heart Centre, Department of Cardiology, Amsterdam Cardiovascular Sciences, Amsterdam, the Netherlands; b HeartLife klinieken, Utrecht, the Netherlands; c Amsterdam UMC, Amsterdam Cardiovascular Sciences, Department of Physiology, Amsterdam UMC, Amsterdam, the Netherlands; d Maastricht University, Cardiovascular Research Institute Maastricht, Department of Physiology, Maastricht, the Netherlands

**Keywords:** ANOCA, Coronary artery spasm, Haemodynamic changes, Acetylcholine, ANOCA, angina and no obstructive coronary artery disease, APV, average peak velocity, CAS, coronary artery spasm, CBF, coronary blood flow, COVADIS, Coronary Vasomotor Disorders International Study Group, ICFT, invasive coronary vasomotor function testing, QCA, quantitative coronary angiography, VSMC, vascular smooth muscle cells

## Abstract

Background: Vasoreactivity testing with high-dose acetylcholine is considered vasospasm provocation and low-dose as endothelial function testing. Aims: To assess the changes in reaction to low- and high-dose acetylcholine in the endotypes of CAS as defined by the Coronary Vasomotor Disorders International Study Group (COVADIS) working group. Methods: Changes in coronary epicardial diameter, coronary blood flow (CBF) and vascular resistance were determined at low-dose acetylcholine. Results: A total of 88 ANOCA patients were included in this analysis. In the negative group (n = 14) incremental infusion of acetylcholine produced a progressive increase in CBF (p = 0.008). In reaction to low-dose acetylcholine, the epicardial vasospasm group (n = 30) is characterised by epicardial vasoconstriction that is significantly more severe compared to the microvascular vasospasm group (p = 0.004)(n = 23). The equivocal group (n = 21) is characterised by an increase in CBF and reduction in vascular resistance that are both significantly different compared to the epicardial vasospasm group (p = 0.036 and p = 0.007, respectively). High-dose acetylcholine decreased epicardial diameter and CBF significantly in the epicardial vasospasm, microvascular vasospasm and in the equivocal group (all p < 0.05. Vascular resistance increased significantly in the epicardial vasospasm group (p < 0.001) and equivocal group (p = 0.009). Conclusion: In reaction to low-dose acetylcholine the negative and equivocal endotype has haemodynamic changes that suggest intact endothelium. In reaction to high-dose acetylcholine the epicardial vasospasm, microvascular vasospasm and equivocal endotype have hemodynamic changes that suggest VSMC-hyperreactivity. These results suggest that the equivocal endotype is a positive test comparable to microvascular vasospasm in the presence of normal endothelial function.

## Highlights

• In reaction to low-dose acetylcholine the negative and equivocal endotype has haemodynamic changes that suggest intact endothelium.
• In reaction to high-dose acetylcholine the epicardial vasospasm, microvascular vasospasm and equivocal endotype have hemodynamic changes that suggest VSMC-hyperreactivity.
• The data of our study shows that the equivocal endotype is a form of microvascular spasm in the presence of a normal endothelial function, and therefore these patients might benefit from treatment with anti-vasospastic medication.

## 1 Introduction

Coronary artery vasospasm (CAS) is an established cardiac condition that may cause anginal complaints in patients without obstructive coronary artery disease (ANOCA) on coronary angiography (CAG). It is caused by a sudden severe narrowing or total occlusion of epicardial and/or microvascular vessels that in turn reduce blood coronary blood flow to the myocardium. This so called vasomotor disorder can be evaluated by an intracoronary acetylcholine vasospasm provocation. The diagnostic criteria for different endotypes of vasomotor disorder are published by the Coronary Vasomotor Disorders International Study Group (COVADIS) and are considered the gold standard for assessment of CAS. Within these diagnostic criteria 3 characteristics are evaluated: (i) the occurrence of the previously reported chest pain,(ii) the induction of ischaemic ECG changes (ST-segment deviation and new U-waves), and (iii) epicardial vasoconstriction of >90 % on CAG [3], [4]. Based on these characteristics the following test results may follow spasm provocation; epicardial vasospasm when all 3 criteria are met, microvascular vasospasm when only the first 2 positive criteria are met, no vasospasm when no criteria are met or an equivocal test result when the first or second criteria is met. Acetylcholine vasoreactivity testing with high-dose acetylcholine is considered as vasospasm provocation test, while with low-dose acetylcholine as a test for coronary endothelial function evaluated by epicardial vasomotion and change in CBF [5], [6], [7]. As such, haemodynamic changes in reaction to low-dose acetylcholine have been extensively described in the literature. In contrast, these changes in reaction to low- and high-dose acetylcholine of the different endotypes of COVADIS in the same patient have not been described previously, while that is relevant to improve the diagnostic utility and interpretation of the acetylcholine intracoronary function test and thereby guide medical treatment (see Table 1).

**Table 1:** Baseline characteristics.

	**Total** (n = 88)	**Negative** (n = 14)	**Epicardial spasm** (n = 30)	**Microvascular spasm** (n = 23)	**Equivocal** (n = 21)	p-value
Age, Y	56.4 ± 10.3	54.4 ± 13.3	59.2 ± 9.4	55.4 ± 9.7	55.38 ± 9.9	0.209
Female, n(%)	76 (87 %)	13 (93 %)	25 (83 %)	22 (96 %)	16 (76 %)	0.234
Height, cm	169.5 ± 8.8	165.7 ± 8.1	169.1 ± 9.4	169.1 ± 6.8	172.3 ± 9.7	0.180
Weight, kg	74.8 ± 13.9	68.8 ± 11.5	77.4 ± 15.2	73.6 ± 11.1	75.1 ± 15.7	0.249
BMI, kg/m^2^	25.9 ± 4.2	25.2 ± 4.8	26.9 ± 4.4	25.5 ± 3.3	25.5 ± 4.7	0.489

**Coronary risk factors**						
Hypertension, n(%)	43 (49 %)	5 (36 %)	15 (50 %)	12 (53 %)	11 (52 %)	0.756
Hypercholesterolemia, n(%)	32 (37 %)	5 (36 %)	15 (50 %)	8 (35 %)	4 (19 %)	0.119
Diabetes, n(%)	5 (6 %)	0 (0 %)	1 (3 %)	3 (13 %)	1 (5 %)	0.319
Current smoking, n(%)	6 (7 %)	1 (6 %)	4 (14 %)	0 (0 %)	1 (5 %)	0.570
Past smoking, n(%)	25 (29 %)	3 (21 %)	7 (24 %)	8 (33 %)	7 (33 %)	
Family history of CVD, n(%)	65 (74 %)	11 (79 %)	19 (63 %)	19 (83 %)	16 (76 %)	0.659
Normal left ventricular ejection fraction (>55 %), n(%)	86/88 (98 %)	12 (92 %)	28 (97 %)	23 (100 %)	21 (100 %)	0.414
Obstructive Coronary artery disease (>50 % stenosis)	0 (0 %)	0 (0 %)	0 (0 %)	0 (0 %)	0 (0 %)	NA
Clinical complaints						
Predominantly resting angina	34 (39 %)	6 (43 %)	8 (27 %)	10 (44 %)	10 (48 %)	0.414
Predominantly effort angina	8 (9 %)	1 (7 %)	2 (7 %)	2 (9 %)	3 (14 %)	0.809
Both resting and effort angina	46 (52 %)	7 (50 %)	20 (67 %)	11 (48 %)	8 (38 %)	0.222

**Acetylcholine provocation**						
Induction of recognisable angina		0 (0 %)	34 (100 %)	27 (100 %)	20 (95 %)	
ECG changes (COVADIS)		0 (0 %)	34 (100 %)	27 (100 %)	1 (5 %)	
Epicardial lumen reduction of ≥ 90 % (as assessed by QCA)		0 (0 %)	34 (100 %)	0 (0 %)	9 (43 %)	

**Adenosine testing**						
CFR	3.2 ± 0.8	3.2 ± 0.8	3.1 ± 0.8	3.4 ± 0.8	3.3 ± 0.8	0.457
HMR	1.9 ± 0.6	1.8 ± 0.8	1.8 ± 0.6	1.9 ± 0.4	2.1 ± 0.5	0.387

One-way ANOVA analyses of baseline characteristic between the COVADIS diagnostic endotypes that follow acetylcholine vasospasm provocation testing. BMI: Body Mass Index, CVD: Cardiovascular disease, COVADIS: The Coronary Vasomotor Disorders International Study Group, QCA: Quantitative Coronary Angiography, CFR: Coronary Flow Reserve, HMR: hyperaemic microvascular resistance.

## 2 Methods

### 2.1 Study design

ANOCA patients who underwent clinically indicated comprehensive invasive acetylcholine intracoronary function testing at the Amsterdam UMC – location Academic Medical Center (AMC) (Amsterdam, The Netherlands) between November 2016 and July 2020 were included in this retrospective cohort study. Ethical approval for this study was waived by the Medical Ethics Review Committee of the AMC as the procedures were performed as part of routine clinical care.

### 2.2 Procedure and data acquisition

Diagnostic CAG was performed using standard techniques, although without the use of radial cocktail or intracoronary nitrates prior to acetylcholine infusions. Beta blockers were discontinued for at least 72 h and all other vasoactive medication for at least 24 h prior to testing.

After diagnostic CAG, a 0.014-inch guidewire equipped with a Doppler crystal (ComboWire XT, Philips Volcano Corporation, San Diego, CA) was advanced into the proximal or mid left anterior descending coronary artery to allow continuous registration of average peak flow velocity (APV). In some of the cases, a microcatheter was used to stabilize the Doppler flow signal. Aortic pressure (Pa) was continuously measured through the guiding catheter.

**Fig. 1:**
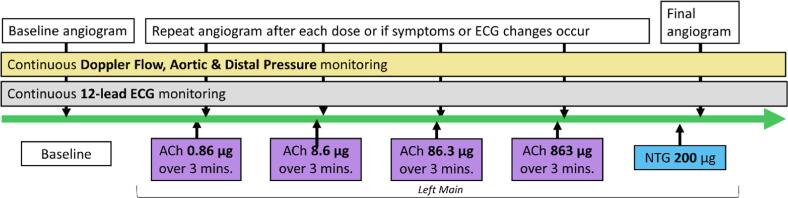
Flow chart of study protocol.

Acetylcholine intracoronary function testing consisted of four incremental doses of 0.86 µg (dose 1), 8.63 µg (dose 2), 86.3 µg (dose 3) and 863 µg (dose 4) infused in 3 min using a mechanical pump. For the purpose of this analysis we excluded patients with a vasospastic reaction to the first three infusions. Therefore, the first three infusions are considered low-dose and the fourth infusion as high-dose, functioning as endothelial function test and provocation test respectively.[5], [6], [7] After acetylcholine intracoronary function testing 200 µg of nitro-glycerine was administered intracoronary (see Fig. 1). A 12-lead ECG was continuously recorded using Mac-Lab (GE, United States).

### 2.3 Diagnostic criteria

Definitions and diagnostic criteria for CAS were defined according to the definitions used by the COVADIS working group [8]. For the diagnosis of epicardial vasospasm all of the following are required in reaction to acetylcholine; (i) a reproduction of the previously reported angina, (ii) the induction of ischaemic ECG changes (ST-segment deviation or new U-waves), and (iii) > 90 % epicardial vasoconstriction by visual assessment of the entire coronary tree [4], [8]. Microvascular vasospasm occurred when the first two criteria mentioned above are met although in absence of epicardial vasoconstriction of >90 % [8]. The test result was considered equivocal when only angina or ECG changes occurred. The test was considered negative when neither recognisable angina nor ECG changes occurred.

### 2.4 Angiography and haemodynamic signal analysis

Quantitative CAG analysis was performed offline (QCA-CMS version 7.3, MEDIS Medical, Leiden, The Netherlands) from a singular angiogram at the end diastolic phase to determine the coronary diameter at a fixed distance 5 mm from the tip of the Doppler crystal that was placed in the proximal or mid LAD. All analysis were performed by RF and independently by CB, JW and CV whom were unaware of the result of the test and the haemodynamic data. Measurements that differed >15 % were independently verified by a senior operator (MB).

Epicardial diameter change was assessed at the end diastolic phase and 5 mm from the tip of the Doppler crystal. Haemodynamic data was extracted from the digital archive (ComboMap, Philips-Volcano, San Diego, CA) and analysed offline using custom software written in Matlab (Mathworks, Inc, Natick, MA). Five-beat averages of instantaneous peak velocity (APV) and Pa were determined at baseline before acetylcholine infusion and at the end of each intracoronary infusion of acetylcholine. Coronary blood flow (CBF) was calculated as π (average peak velocity/2) (vessel diameter/2)^2^ and coronary vascular resistance as the ratio of Pa to CBF.

### 2.5 Haemodynamic changes to low-dose and high-dose acetylcholine

We differentiated the haemodynamic changes that occurred as a result of low-dose (dose 1 to 3) and high-dose (dose 4) acetylcholine administration. In some patients low dose acetylcholine induced a vasospastic reaction that precluded further administration of acetylcholine and were therefore excluded from this analysis. The haemodynamic changes due to low-dose acetylcholine were determined at the third dose compared to baseline as is common in coronary endothelial function testing, which reflects the maximal change in CBF due to endothelial dependent vasodilation [9]. The cumulative effect of high-dose acetylcholine was calculated as the haemodynamic changes in reaction to the fourth dose of acetylcholine compared to third dose (e.g. Coronary Flow Reserve dose 4 – Coronary Flow Reserve dose 3). This allowed to differentiate the change in the haemodynamic parameters due to vasoconstriction or VSMC hyperreactivity from endothelial dependent vasodilation [24].

### 2.6 Statistical analysis

Normality and homogeneity of variances were tested using Shapiro-Wilk and Levene tests. All hemodynamic data are expressed as mean ± standard deviation in text and as mean ± standard error of the mean (SEM) in figures. All other continuous variables are presented as mean ± standard deviation (SD) or median (first, third quartile [Q1, Q3]), and were compared with Student *t* test or Mann-Whitney *U* test, as appropriate. Categorical variables are presented as counts and percentages, and were compared using Fisher exact test.

The effect of incremental infusions of acetylcholine within diagnostic endotype were assessed with a repeated measures ANOVA using change in vascular response (CBF, coronary vascular resistance and epicardial diameter change) as the main factor and dose acetylcholine as repeated measure variable.

Hemodynamic changes as a result of low-dose and high-dose acetylcholine were compared among the different endotypes by using an unpaired Student's *t*-test. Finally, hemodynamic changes due to the effects of low- and high-dose acetylcholine were compared within different endotypes with a paired *T*-test. All statistical analyses were performed using SPSS version 27 (IBM Corp., Armonk, NY, USA). *P* < 0.05 was considered statistically significant.

## 3 Results

### 3.1 Study population

**Fig. 2:**
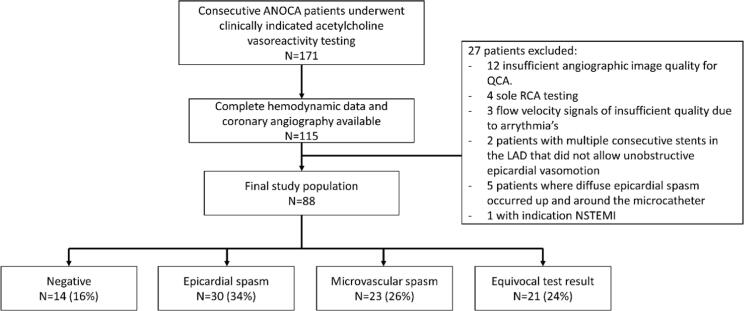
Flowchart of the final study population and diagnosis after exclusions.

A total of 171 consecutive patients underwent clinically indicated acetylcholine intracoronary function test, of whom 115 patients (67 %) received all four doses of acetylcholine, and had complete hemodynamic and coronary angiography data available for the purpose of this analysis. From these, 27 patients (23 %) were excluded from analysis for reasons outlined in Fig. 2. The final study population comprised 88 patients and was predominantly female (87 %), with a mean age of 56.4 ± 10.3 years.

### 3.2 Results of acetylcholine intracoronary function test

Acetylcholine intracoronary function testing revealed that 16 % (n = 14) of patients had a negative test result as no recognisable angina and/or ischaemic ECG changes could be induced (Fig. 2). A further, 34 % (n = 30) tested positive for epicardial vasospasm, 26 % (n = 23) for microvascular vasospasm and 24 % (n = 21) of patients had an equivocal test result. In the equivocal group spasm, 20 patients had recognisable angina without ECG changes and 1 patient had ECG changes without recognisable anginal complaints. Demographic and clinical data of the total population are summarised in Table 1.

### 3.3 Haemodynamic changes in reaction to incremental intracoronary acetylcholine administration

**Fig. 3:**
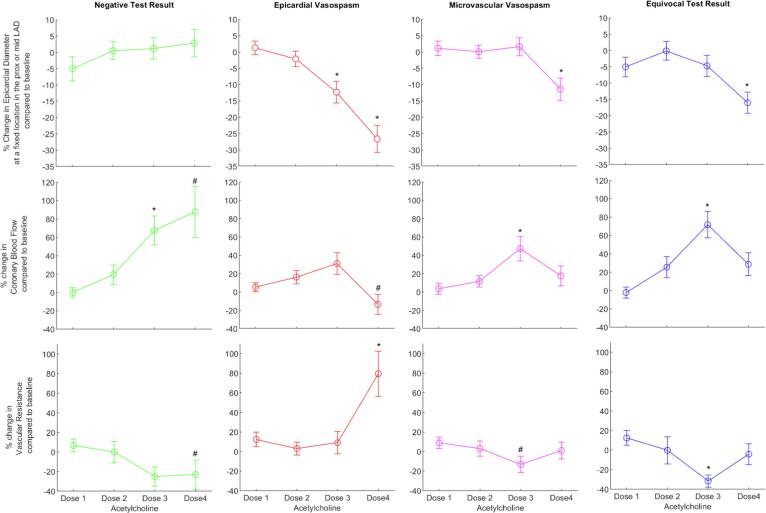
Dose response relationship of patients who completed the entire ICFT protocol. Acetylcholine spasm provocation testing consisted of four incremental doses in three minute infusion of acetylcholine concentrations of 0.2 µg/ml (dose 1), 2.1 µg/ml (dose 2), 21.1 µg/ml (dose 3) and 211 µg/ml (dose 4) infused at 82 ml/h using a mechanical pump. * indicates p < 0.05 after repeated measure ANOVA and # indicates that this response was non-normally distributed and p < 0.05 after Wilcoxon signed rank test. All variable that tested p < 0.05 after repeated measure ANOCA were also significant when tested non-parametrically.

The changes in coronary epicardial diameter measured at a fixed location in the proximal or mid LAD, CBF and coronary vascular resistance compared to baseline in response to incremental doses of acetylcholine among the diagnostic endotypes are presented in Fig. 3. In the negative group (reference group) incremental intracoronary infusion of acetylcholine produced a progressive increase in CBF (p = 0.008), whereas the changes in epicardial diameter and vascular resistance were non-significant.

**Fig. 4:**
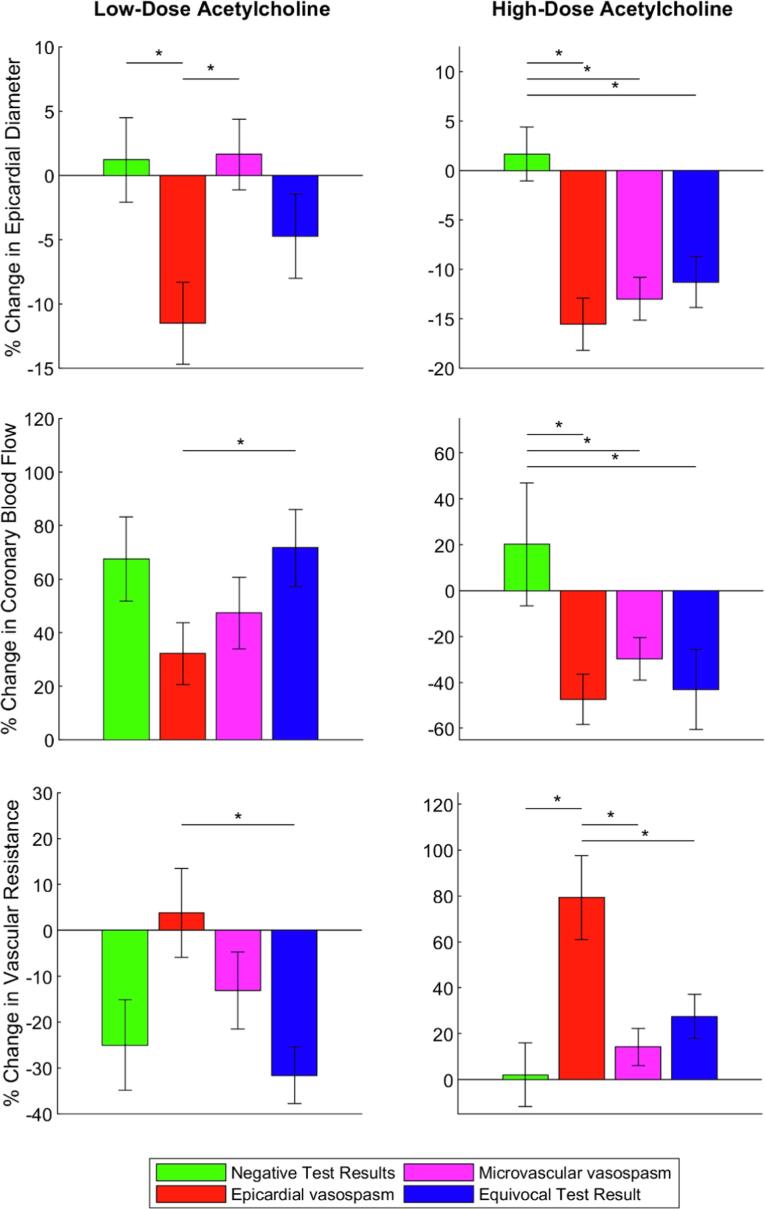
Bar chart comparing haemodynamic changes (epicardial diameter, coronary blood flow and vascular resistance) to low- and high-dose acetylcholine among the diagnostic endotypes according to the diagnostic criteria of the COVADIS workgroup. Haemodynamic changes to low-dose are calculated as the ratio of haemodynamic changes in reaction to dose 3 to baseline. Hemodynamic changes as a result of high-dose acetylcholine were calculated by subtracting the haemodynamic changes in reaction to the third dose of acetylcholine compared to baseline from the haemodynamic changes in reaction to the fourth dose of acetylcholine compared to baseline (e.g. Coronary Flow Reserve dose 4 – Coronary Flow Reserve dose 3). * indicates p < 0.05 after T-test comparison with the negative test result.

The haemodynamic changes in reaction to low-dose acetylcholine (dose 1 to 3) compared to baseline per diagnostic endotype is shown in Fig. 3 and Fig. 4, left panel. The epicardial vasospasm group is characterised by gradual decrease in epicardial diameter measured at a fixed location in the proximal or mid LAD in reaction to incremental doses of acetylcholine (dose 3; p = 0.011), by a non-significant increase in CBF and by no significant change in vascular resistance. In the microvascular vasospasm group CBF increases in reaction to the low-dose acetylcholine (does 3; p = 0.017), whilst epicardial diameter and vascular resistance did not change significantly. The equivocal endotype is characterised by a significant increase in CBF (dose 3; p < 0.001) and a significant decrease in vascular resistance in reaction (dose 3; p < 0.001) to the low dose of acetylcholine, whilst epicardial diameter remained unchanged.

The haemodynamic changes in reaction to high-dose acetylcholine (dose 4) compared to baseline per diagnostic endotype is shown in Fig. 3. The epicardial vasospasm group is characterised by a decrease in epicardial diameter as a result of epicardial vasospasm in reaction to the highest dose of acetylcholine (dose 4; p < 0.001), a non-significant decrease in CBF and by a marked increase in vascular resistance (dose 4; p = 0.023). In the microvascular vasospasm group the epicardial diameter decreased significantly (dose 4; p = 0.016), while CBF and vascular resistance increased non-significantly in reaction to the highest dose of acetylcholine. The equivocal endotype is characterized by a significant reduction in epicardial diameter in reaction to the highest dose of acetylcholine (dose 4; p < 0.001). There were no significant changes in CBF and in vascular resistance compared to baseline.

### 3.4 Cumulative effect of high-dose after low-dose

In Fig. 4, right panel, the haemodynamic changes of high-dose acetylcholine is expressed as the cumulative effect of the fourth dose after the third dose (e.g. Coronary Flow Reserve dose 4 – Coronary Flow Reserve dose 3). Changes in epicardial diameter measured at a fixed location in the proximal to mid LAD, CBF and vascular resistance in reaction to the third and fourth dose acetylcholine did not differ significantly in the negative group (p = 0.551, p = 0.466 and p = 0.890, respectively). In the epicardial vasospasm group change in epicardial diameter, CBF and vascular resistance differed significantly (all p < 0.001). In the microvascular vasospasm group epicardial diameter response and CBF change were significantly different (p < 0.001, p = 0.004, respectively), while that of vascular resistance only numerically (p = 0.093). The equivocal test result group also had a significant different response to the third dose compared to the fourth as change in epicardial diameter, CBF and vascular resistance were significantly different (p < 0.001, p = 0.023 and p = 0.009, respectively).

### 3.5 Comparison between different endotypes

The change in epicardial diameter in the epicardial vasospasm group in reaction to low-dose acetylcholine is significantly reduced compared to the negative test result (p = 0.019) and the microvascular vasospasm group (p = 0.004). The increase in CBF and reduction in vascular resistance in the equivocal test result group were both significantly different compared to the epicardial vasospasm group (p = 0.036 and p = 0.007, respectively).

The change in epicardial diameter in the epicardial vasospasm group, microvascular vasospasm group and equivocal group in reaction to high-dose acetylcholine was significantly different compared to the negative test result (p < 0.001, p < 0.001 and p = 0.002, respectively). A similar difference between the negative endotype and all other endotypes was seen in the change in CBF, where in the negative test result no change in CBF occurred versus a decreased CBF in the epicardial vasospasm, microvascular vasospasm and equivocal test result (p = 0.006, p = 0.045 and p = 0.046, respectively). Vascular resistance was significantly increased in the epicardial vasospasm group compared to the negative, microvascular vasospasm and equivocal test result. (p = 0.002, p = 0.002 and p = 0.016, respectively).

## 4 Discussion

This study showed that there are distinct differences in haemodynamic changes in response to low- and high-dose acetylcholine among the diagnostic endotypes of vasospasm provocation defined by the COVADIS diagnostic criteria:

(1) The negative test result is characterised by a dose-dependent increase in CBF as a result of intact endothelium. Furthermore, haemodynamic changes in response to the low dose of acetylcholine do not differ from those that occur at the high dose, in contrast to all other diagnostic endotypes suggesting the absence of a vasospastic response.
(2) The epicardial vasospasm group is characterised by a significant epicardial diameter reduction and a diminished increase in CBF (<50 %) in reaction to low-dose acetylcholine. A decrease in CBF and increase in vascular resistance is observed at high dose acetylcholine.
(3) The microvascular vasospasm group is characterised by a diminished endothelial function resulting in a < 50 % increase in CBF to low-dose acetylcholine and by a significant epicardial vasoconstriction after high dose acetylcholine.
(4) The equivocal test result is characterised by a normal endothelial function as CBF increases > 50 % and is the only endotype in which low-dose acetylcholine induced a significant reduction in vascular resistance. Interestingly, haemodynamic changes to high dose acetylcholine suggest vasospasm similar to haemodynamic changes that occur in the microvascular vasospasm group.

### 4.1 Haemodynamic changes to the low dose of intracoronary acetylcholine

In reaction to low-dose acetylcholine administration two distinct reactions can be described: 1) vasodilation by stimulating the endothelium to produce vasodilatory substances and 2) a vasoconstriction through direct VSMC activation. In the presence of healthy endothelium the vasodilatory component outbalances the vasoconstrictive component resulting in coronary haemodynamic changes that suggest epicardial and microvascular vasodilation. If the endothelium is diseased, the extent of vasodilation can be reduced, absent, or some vasoconstriction can be induced.

In clinical practice, an abnormal coronary endothelial function is considered when CBF increases < 50 % and/or epicardial vasoconstriction occurs in reaction to low-dose acetylcholine compared to baseline [10], [11], [12]. Coronary endothelial dysfunction is associated with impaired quality of life as well as a high incidence of adverse cardiovascular events [10], [13], [14], [15], [16]. In our study, the equivocal group was characterised by an increase in CBF (>50 %) and a reduction in vascular resistance that were both significant indicative of the presence of intact endothelium. Noteworthy, the response in epicardial diameter and CBF in the equivocal group was significantly different from the epicardial vasospasm group.

### 4.2 Haemodynamic changes to the high dose of intracoronary acetylcholine

In CAS vascular smooth muscle cells (VSMC) hyperreactivity is thought to be the pathophysiological substrate which can be reproduced in the catheterisation laboratory with the administration of high doses of acetylcholine [5], [17]. This hyperreactive response is reflected in the haemodynamic changes (reduction of epicardial diameter and CBF and/or an increase in vascular resistance) that are known to occur in the epicardial and microvascular vasospasm groups. These changes in epicardial diameter and CBF are significantly different compared to that of the negative group. In the negative diagnostic endotype haemodynamic changes in reaction to high-dose did not differ significantly from those that occur at low-dose; e.g. coronary epicardial diameter remained on averaged unchanged, CBF remained increased and coronary vascular resistance remained reduced. This suggests that even at high-dose acetylcholine endothelium effectuated vasodilation outbalances vasoconstriction through direct VSMC stimulation in the absence of a VSMC hyperreactive response.

The haemodynamic changes as in response to high-dose acetylcholine in the equivocal group mirrors that of the microvascular spasm group; e.g. significant reduction in epicardial diameter and CBF that are also significantly different from of the negative test result. The fact that a vasospastic response occurs in the equivocal group in the presence of an intact endothelial function, suggests that vasospasm can occur irrespectively of endothelial function. This is in line with a previous study where substance P, a purely endothelium dependent vasodilatory substance, was used to measure endothelial function in patients with epicardial vasospasm. They found that this substance produced a comparable dose dependent vasodilatory response at the vasospastic site as to the non-vasospastic sites [18].

Our data show that in the microvascular group ischemia may occur despite minimal changes in CBF and VR compared to baseline as measured in the epicardial segment. That might be explained by a heterogenous response at the microcirculatory level where regions of myocardial ischemia induced by vasospasm that are compensated by ACh mediated vasodilation in other regions that may result in a minimal change in CBF and VR as assess at the epicardial level.

### 4.3 Implications for acetylcholine intracoronary function testing

The diagnostic criteria for the diagnosis of CAS published by the COVADIS working group are widely accepted and applied in current clinical practise and clinical trials [19]. These criteria state that ECG changes are necessary as an objective measure of ischemia in addition to inducible recognisable angina in reaction to acetylcholine [4], [8]. Although, one of the havocs of these criteria is the occurrence of equivocal test results in which not all criteria for a positive test are met. This group poses a diagnostic and therapeutic dilemma as these patients are considered negative according to the COVADIS criteria, whilst in fact this group shows coronary haemodynamic changes at the vasospastic dose similar to the microvascular spasm endotype [20], [21]. In the current study population 24 % of patients undergoing spasm provocation have such an equivocal test result, of which almost all due to inducible angina without the necessary ECG changes (96 %). The percentage of equivocal test result in the current study is similar to that reported previously; 29 % of 1379 patients undergoing spasm provocation in the ACOVA study and among 20.1 % in a Korean cohort of 4644 patients by Lee et al. [20], [21]. Results of the latter study suggests that the equivocal diagnostic endotype is a form of vasospasm as these patients had similar features to epicardial vasospasm, such as a male dominance and a more frequently observed fixed lesion on baseline CAG as opposed to other diagnostic endotypes. Moreover, on follow-up of both studies there was no difference in recurrent angina, Seattle angina score, between the equivocal, microvascular and epicardial spasm group, in contrast to patients with a normal test result [21]. The results of the current study adds on the premise that the equivocal endotype is a form of vasospasm [20], [21]. Such an equivocal test result might be due to the presence of a normal endothalial function of that could parltly offset acetylcholine induced vasospasm. Alternatively, an equivocal test result may be more prevelant in patients in whom the size of the ischemic region caused by vasospasm is relatively small. Consequently sufficient electrocardiographic changes do not appear, and as a result, if only chest pain is provoked, it may be diagnosed as an equivocal test result.

### 4.4 Clinical implications

Current recommendations of the COVADIS working group do not recommend treatment of patients with a equivocal test result, which may lead to a worse cardiovascular prognosis, persistent angina, and a reduced quality of life [22], [23]. The data of our study challenges this recommendation as an equivocal endotype is a form of microvascular spasm in the presence of a normal endothelial function, and therefore these patients might benefit from treatment with anti-vasospastic medication. In contrast to microvascular vasospasm and epicardial vasospasm endotypes, the equivocal diagnostic endotype characterized by an intact endothelium would presumably less likely benefit from treatment with endothelium modifying drugs such as ace-inhibitors. We believe that a diagnostic reclassification of the equivocal endotype in the COVADIS working group recommendations is appropriate and that treatment with medication should be recommended. Further randomized controlled trails are needed to establish optimal treatment in the equivocal endotype.

## 5 Limitations

There a several limitations of this study that need to be addressed. Firstly, this was a retrospectively study with small number of predominantly female patients. Nevertheless, such detailed information on coronary haemodynamic changes that occur in reaction to acetylcholine has not been reported on before. Secondly, medication was withheld for 24 h and therefore the effect of long acting calcium antagonist cannot be excluded.

Thirdly, the analysis has been performed on generalisation of diagnostic endotypes based on COVADIS diagnostic criteria, which inherently have their own limitations. Lastly, due to the designs of the study there is no follow-up of patients with the equivocal endotype which could prove that these patients would indeed. Future studies could provide this information.

## 6 Conclusion

In reaction to low-dose acetylcholine the negative and equivocal test result have hemodynamic changes that suggest intact endothelium, whereas in the epicardial and microvascular vasospasm group show endothelial dysfunction. In reaction to high-dose acetylcholine the epicardial vasospasm, microvascular vasospasm and equivocal test result group have hemodynamic changes that suggest VSMC hyperreactivity in contrast to the negative test result. The results of this study demonstrate that patients with an equivocal test result have haemodynamic characteristics that are similar to patients with microvascular vasospasm, although in the presence of a normal endothelial function. These findings warrant further investigation regarding the optimal treatment strategy in patients with an equivocal test result.

## Declaration of Competing Interest

The authors declare that they have no known competing financial interests or personal relationships that could have appeared to influence the work reported in this paper.

## References

[3] Group JCSJW. Guidelines for diagnosis and treatment of patients with vasospastic angina (Coronary Spastic Angina) (JCS 2013). Circ J. 2014;78(11):2779-801. 10.1253/circj.cj-66-0098 25273915

[4] Ong P., Camici P.G., Beltrame J.F., Crea F., Shimokawa H., Sechtem U., Kaski J.C., Bairey Merz C.N. (2018). Coronary vasomotion disorders international study G. International standardization of diagnostic criteria for microvascular angina. Int. J. Cardiol..

[5] Okumura K., Yasue H., Matsuyama K., Goto K., Miyag H., Ogawa H., Matsuyama K. (1988). Sensitivity and specificity of intracoronary injection of acetylcholine for the induction of coronary artery spasm. J. Am. Coll. Cardiol..

[6] Lerman A., Holmes D.R., Bell M.R., Garratt K.N., Nishimura R.A., Burnett J.C. (1995). Endothelin in coronary endothelial dysfunction and early atherosclerosis in humans. Circulation.

[7] Feenstra R.G.T., Seitz A., Boerhout C., Bukkems L.B., Stegehuis V.S., Teeuwisse P.T., de Winter R.J., Sechtem U., Piek J.J., van de Hoef T.P., Ong P., Beijk M.A.M. (2022). Principles and pitfalls in coronary vasomotor function testing. EuroIntervention..

[8] Beltrame J.F., Crea F., Kaski J.C., Ogawa H., Ong P., Sechtem U., Shimokawa H., Bairey Merz C.N. (2017). Coronary Vasomotion Disorders International Study G. International standardization of diagnostic criteria for vasospastic angina. Eur. Heart J..

[9] Corban M.T., Godo S., Burczak D.R., Noseworthy P.A., Toya T., Lewis B.R., Lerman L.O., Gulati R., Lerman A. (2020). Coronary endothelial dysfunction is associated with increased risk of incident atrial fibrillation. J. Am. Heart Assoc..

[10] Suwaidi J.A., Hamasaki S., Higano S.T., Nishimura R.A., Holmes D.R., Lerman A. (2000). Long-term follow-up of patients with mild coronary artery disease and endothelial dysfunction. Circulation.

[11] Hasdai D., Gibbons R.J., Holmes D.R., Higano S.T., Lerman A. (1997). Coronary endothelial dysfunction in humans is associated with myocardial perfusion defects. Circulation.

[12] Wei J., Mehta P.K., Johnson B.D., Samuels B., Kar S., Anderson R.D., Azarbal B., Petersen J., Sharaf B., Handberg E., Shufelt C., Kothawade K., Sopko G., Lerman A., Shaw L., Kelsey S.F., Pepine C.J., Merz C.N. (2012). Safety of coronary reactivity testing in women with no obstructive coronary artery disease: results from the NHLBI-sponsored WISE (Women's Ischemia Syndrome Evaluation) study. JACC Cardiovasc Interv..

[13] Lerman A., Zeiher A.M. (2005). Endothelial function: cardiac events. Circulation.

[14] Halcox J.P., Schenke W.H., Zalos G., Mincemoyer R., Prasad A., Waclawiw M.A., Nour K.R., Quyyumi A.A. (2002). Prognostic value of coronary vascular endothelial dysfunction. Circulation.

[15] Schächinger V., Britten M.B., Zeiher A.M. (2000). Prognostic impact of coronary vasodilator dysfunction on adverse long-term outcome of coronary heart disease. Circulation.

[16] von Mering G.O., Arant C.B., Wessel T.R., McGorray S.P., Bairey Merz C.N., Sharaf B.L., Smith K.M., Olson M.B., Johnson B.D., Sopko G., Handberg E., Pepine C.J., Kerensky R.A., National Heart L., Blood I. (2004). Abnormal coronary vasomotion as a prognostic indicator of cardiovascular events in women: results from the National Heart, Lung, and Blood Institute-Sponsored Women's Ischemia Syndrome Evaluation (WISE). Circulation.

[17] Hubert A., Seitz A., Pereyra V.M., Bekeredjian R., Sechtem U., Ong P. (2020). Coronary artery spasm: the interplay between endothelial dysfunction and vascular smooth muscle cell hyperreactivity. Eur Cardiol..

[18] Kensuke Egashira T.I., Yamada A., Hirooka Y., Takeshita A. (1992). Preserved endothelium-dependent vasodilation at the vasospasctic site in patients with variant angina. J. Clin. Investigat..

[19] Kunadian V., Chieffo A., Camici P.G., Berry C., Escaned J., Maas A., Prescott E., Karam N., Appelman Y., Fraccaro C. (2020). An EAPCI expert consensus document on ischaemia with non-obstructive coronary arteries in collaboration with European society of cardiology working group on coronary pathophysiology & microcirculation endorsed by coronary vasomotor disorders international study group. Eur. Heart J..

[20] Ong P., Athanasiadis A., Borgulya G., Vokshi I., Bastiaenen R., Kubik S., Hill S., Schaufele T., Mahrholdt H., Kaski J.C., Sechtem U. (2014). Clinical usefulness, angiographic characteristics, and safety evaluation of intracoronary acetylcholine provocation testing among 921 consecutive white patients with unobstructed coronary arteries. Circulation.

[21] Lee E.M., Choi M.H., Seo H.S., Kim H.K., Kim N.H., Choi C.U., Kim J.W., Lim H.E., Kim E.J., Rha S.W., Park C.G., Oh D.J. (2017). Impact of vasomotion type on prognosis of coronary artery spasm induced by acetylcholine provocation test of left coronary artery. Atherosclerosis..

[22] Jespersen L., Abildstrøm S.Z., Hvelplund A., Prescott E. (2013). Persistent angina: highly prevalent and associated with long-term anxiety, depression, low physical functioning, and quality of life in stable angina pectoris. Clin Res Cardiol..

[23] Radico F., Zimarino M., Fulgenzi F., Ricci F., Di Nicola M., Jespersen L., Chang S.M., Humphries K.H., Marzilli M., De Caterina R. (2018). Determinants of long-term clinical outcomes in patients with angina but without obstructive coronary artery disease: a systematic review and meta-analysis. Eur. Heart J..

[24] Ohba K., Sugiyama S., Sumida H., Nozaki T., Matsubara J., Matsuzawa Y., Konishi M., Akiyama E., Kurokawa H., Maeda H., Sugamura K., Nagayoshi Y., Morihisa K., Sakamoto K., Tsujita K., Yamamoto E., Yamamuro M., Kojima S., Kaikita K., Tayama S., Hokimoto S., Matsui K., Sakamoto T., Ogawa H. (2012). Microvascular coronary artery spasm presents distinctive clinical features with endothelial dysfunction as nonobstructive coronary artery disease. J Am Heart Assoc..

